# Multidimensional Predictors of Functional Recovery After Curative Colon Cancer Surgery: A Patient-Reported Outcomes Study

**DOI:** 10.7759/cureus.108760

**Published:** 2026-05-12

**Authors:** Maham Nuzhat Lodi, Ahmed Gerwash, Muhammad Amad Afridi, Sheeza Nadeem, Kaushalendra Mani Tripathi, Ieman Zahid, Mohammed Shihas, Majd Aldin Oudeh, Mohamed Anwar Mohamed Ibrahim, Bayan Ahmad Mohammad Rawadieh, Aseel Abubakr Eltigani Elhaj

**Affiliations:** 1 Emergency Medicine, York Hospital, York, GBR; 2 Colorectal Surgery, University Hospital Lewisham, London, GBR; 3 Medicine, Ayub Medical College, Abbottabad, PAK; 4 General Surgery, York Hospital, York, GBR; 5 Medicine, Jinnah Medical and Dental College, Karachi, PAK; 6 Medicine, Doncaster and Bassetlaw Teaching Hospitals NHS Foundation Trust, Doncaster, GBR; 7 Medicine, White River Medical Center-White River Health, Batesville, USA; 8 Medicine and Surgery, Combined Military Hospital, Lahore, PAK; 9 Internal Medicine, Aster DM Healthcare, Dubai, ARE; 10 Medicine, Department of Health (DOH), Abu Dhabi, ARE; 11 Medicine, Odessa National Medical University, Odessa, UKR; 12 Medicine and Surgery, University of Gezira, Wad Madani, SDN; 13 Medicine and Surgery, Prince Hamzah Hospital, Amman, JOR; 14 Surgery, Al Qassimi Hospital, Sharjah, ARE

**Keywords:** colon cancer, comorbidities, curative resection, functional status, quality of life

## Abstract

Background: Functional status, comorbidities, and quality of life (QoL) are patient-related factors that affect QoL outcomes following curative surgery for colon cancer, although limited information exists on these factors in low- and middle-income nations.

Materials and methods: It was a cross-sectional, questionnaire-based study involving 402 adults who underwent curative resection in public and private hospitals in Pakistan because of colon cancer. The Eastern Cooperative Oncology Group (ECOG) Performance Status was used to assess functional status, the Charlson Comorbidity Index (CCI) to assess comorbidity burden, and the Functional Assessment of Cancer Therapy-Colon (FACT-C) to determine QoL. Correlation analysis, descriptive statistics, one-way analysis of variance (ANOVA), and multiple linear regression were conducted.

Results: There were strong positive correlations among all QoL domains (p < 0.05). There was a significant difference in functional well-being across age groups, with the highest score among participants aged > 60 years (p = 0.006). The smoking condition did not have any significant relationship with QoL results. Multiple linear regression revealed that physical well-being (β = 0.251), emotional well-being (β = 0.335), social well-being (β = 0.188), additional concerns (β = 0.209), and age (β = 0.096) had significant predictive value for functional well-being, explaining 38.4% of the variance.

Conclusion: Physical, emotional, and social factors are significantly associated with functional well-being among patients with colon cancer following curative resection. Routine evaluation of QoL and functional status may help identify patients at risk of poorer postoperative well-being and guide tailored supportive care.

## Introduction

Colon cancer is one of the most common causes of cancer-related morbidity and mortality in the world [1]. Curative resection is the mainstay of treatment for localised disease and provides the prospect of prolonged survival [2]. However, survival outcomes following surgery are not strictly governed by tumour stage or surgical technique, but rather by patient-related factors, especially functional status and comorbidities, which play a critical role in determining postoperative recovery, complication rates, and overall prognosis [3].

The ability to perform activities of daily living, or general physical performance, often denoted as functional status, is vital and reflects physiologic reserve and the capacity to withstand the demands of significant surgery [4]. Several studies have shown that impaired functional status is linked to increased postoperative complications, prolonged hospital stay, and reduced survival in patients with colorectal cancer [5,6]. Similarly, comorbid conditions such as cardiovascular disease, diabetes, and chronic respiratory disorders may have a significant impact on surgical outcome, treatment tolerance, and long-term survival [7].

Despite the well-established role of patient-related factors such as functional status and comorbidities in influencing postoperative outcomes, most existing evidence is derived from high-income settings. There remains limited context-specific data from low- and middle-income countries, including Pakistan, where differences in healthcare infrastructure, access to care, and sociocultural factors may influence postoperative outcomes and patient-reported well-being [8].

This study aims to investigate the relationship between functional status and comorbidities and quality-of-life (QoL) outcomes in patients undergoing curative resection for colon cancer by a cross-sectional and questionnaire-based approach. By identifying significant predictors of postoperative functional outcomes, this research aims to contribute to optimising patient management and survival for this population.

Research gap and novelty

Although patient-centred variables such as functional status and comorbidities have been extensively researched in high-income countries, their applicability and effects may differ in other healthcare systems and sociocultural contexts. In Pakistan and other low- and middle-income settings, differences in access to healthcare, perioperative care, and social support systems may affect postoperative outcomes in ways not comprehensively captured in the current literature.

Moreover, there is limited evidence integrating patient-reported functional status, comorbidity burden, and multidimensional QoL outcomes within this regional context. This study addresses this gap by providing data from a Pakistani population using validated patient-reported outcome measures, thereby offering contextually relevant insights into factors associated with postoperative well-being following curative colon cancer surgery.

Objectives

The primary objective of this study was to assess the association between functional status, comorbidity burden, and postoperative QoL outcomes in patients undergoing curative resection for colon cancer. Secondary objectives included examining the relationships among QoL domains and identifying key factors associated with functional well-being using correlation and multiple regression analyses.

## Materials and methods

Study design and setting

The study was a cross-sectional, questionnaire-based study conducted to determine the impact of functional status and comorbidities on QoL outcomes in patients who had undergone curative resection due to colon cancer. Several public and private hospitals in Pakistan were sampled to recruit a diverse sample of patients.

Structured interviews were conducted during routine outpatient visits or during a stable hospital stay. The questionnaires were administered by trained interviewers using a standardised protocol to minimise interviewer bias and ensure uniformity. The use of convenience sampling may introduce selection bias and limit the generalisability of the findings.

Sample size and sampling technique

The target population was assumed to be infinite. Based on a conservative prevalence estimate (p = 0.5), a 95% confidence level (Z = 1.96), and a 5% margin of error, the sample size calculated was 385 [9]. The sample size was increased to 410 participants to account for potential incomplete responses, and 402 complete data sets were included in the final analysis. A convenience sampling technique was used among eligible patients attending outpatient clinics and inpatient wards.

Inclusion and exclusion criteria

Adults who were 18 years or older, had undergone curative resection for colon cancer, and were clinically stable were eligible. Participants had to provide informed written consent and complete the study questionnaires. Patients with severe cognitive impairment, communication difficulties, or other conditions that prevent them from participating were excluded from the study.

Data collection tools

Data were gathered using a structured questionnaire comprising four major sections: demographic data, functional status, comorbidities, and QoL. The questionnaire combined patient-reported measurements and standardised scales to obtain reliable and reproducible results. The original English versions of the scales were used, and trained bilingual interviewers facilitated participants with limited English proficiency. Although bilingual interviewers assisted participants with limited English proficiency, the use of English-language instruments and the exclusion of individuals with communication difficulties may have limited participation from individuals with lower literacy levels or from rural and socioeconomically disadvantaged backgrounds.

Demographic information

Demographic data were obtained to measure personal and social characteristics that may affect QoL outcomes. Information included age, gender, marital status, level of education, occupation, and time since surgery. This section was created by the research team specifically for this study and was used to provide context for patient-reported outcomes in relation to clinical and social factors.

Eastern Cooperative Oncology Group (ECOG) Performance Status

Functional status was measured using the ECOG Performance Status scale developed by Oken et al. in 1982. The ECOG scale assesses a patient's level of functioning in relation to their ability to care for themselves, perform daily activities, and physical capability. It is rated from 0 (completely active) to 5 (dead), with higher scores reflecting worse functional status. Its simplicity, reproducibility, and high inter-rater reliability (k = 0.85) make the scale extensively applicable [10]. The ECOG Performance Status Scale is in the public domain and was used without modification in this study.

Charlson Comorbidity Index (CCI)

Comorbidity was measured using the CCI, which was established in 1987 by Charlson and others. The CCI is used to measure the presence and severity of 19 medical comorbidities, weighted by their relative risk of mortality. The total scores range from 0 to 37, with higher scores indicating greater comorbidity burden. The CCI is very robust (Cronbach's alpha = .87) and also predictive of postoperative outcomes and long-term survival [11]. The CCI was used in English by all the participants.

Functional Assessment of Cancer Therapy-Colon (FACT-C) questionnaire

The QoL was evaluated using the FACT-C questionnaire, developed by Cella et al. in 1993. FACT-C is a tumour-specific instrument having 36 questions assessing four domains, namely, physical well-being, social/family well-being, emotional well-being, and functional well-being, and a colon-specific subscale that evaluates symptoms associated with the disease and treatment. The ratings are given on a five-point Likert scale, with the lowest score of 0 (not at all) to the highest score of 4 (very much); the higher the rating, the better the QoL. The FACT-C has high internal consistency (Cronbach's alpha = 0.89092) and construct validity across different populations [12]. In this research, the English version was employed, with trained interviewers conducting the interviews to ensure clarity and accuracy.

Procedure

Follow-up visits or inpatient visits were conducted with the participants. The data collection was conducted by self-administered questionnaires or structured interviews with trained research assistants after written informed consent was obtained. The entire process was conducted in accordance with privacy and confidentiality, and participants were notified that participation was optional and could be withdrawn at any time.

Statistical analysis

IBM SPSS Statistics for Windows, Version 26 (Released 2018; IBM Corp., Armonk, New York, United States) was used to analyse data. The demographic characteristics, functional status, comorbidities, and QoL were summarised using descriptive statistics, including frequencies and percentages. The Kolmogorov-Smirnov and Shapiro-Wilk tests were used to assess the normality of continuous variables. Although these tests indicated significant deviations from normality and some variables exhibited substantial positive skewness, parametric tests (t-tests, analysis of variance (ANOVA), and multiple linear regression) were retained, given the large sample size (N = 402) and the robustness of these methods under the central limit theorem. However, given the degree of skewness observed in certain variables, the findings should be interpreted with caution. Correlation analysis, t-tests, ANOVA, and multiple regression analyses were used to examine associations between functional status, comorbidity burden, and QoL outcomes, as appropriate. Statistical significance was set at p < 0.05. Cases with incomplete responses were excluded from the final analysis, and only complete datasets (N = 402) were included.

Ethical considerations

The Institutional Review Board of Jinnah Sindh Medical University (JSMU/IRB/ONC/2025/10/014) approved the study protocol, and all procedures used conformed to ethical standards of human research. Before data collection, the participants were asked to provide written informed consent. Data were anonymised and stored securely to maintain confidentiality.

## Results

Table 1 indicates that the largest proportion of participants was in the 41-50 years age group (N = 229, 57.0%), and the majority were females (N = 216, 53.7%). Most of them were married (N = 302, 75.1%) and had either primary or secondary education (N = 363, 90.3%). Over half of them were unemployed (N = 207, 51.5%). The highest number was former smokers (N = 169, 42.0%). The majority of the participants had surgery on curative colon cancer in the recent past (N = 281, 69.9%). The minority reported chronic medical conditions (N = 106, 26.4%), prior major surgery (N = 144, 35.8%), or a family history of cancer (N = 159, 39.6%).

**Table 1:** Demographic characteristics of participants (N = 402) f: frequency; %: percentage Values are presented as N (%); N = 402. No statistical comparisons were performed for demographic variables in this table

Variable	f (N)	%
Age group	-	-
≤40 years	71	17.7
41-50 years	229	57.0
51-60 years	72	17.9
>60 years	30	7.5
Gender	-	-
Male	186	46.3
Female	216	53.7
Marital status	-	-
Single	74	18.4
Married	302	75.1
Widowed/divorced	26	6.5
Educational level	-	-
No formal education	39	9.7
Primary/secondary	183	45.5
Graduate or above	180	44.8
Employment status	-	-
Employed	146	36.3
Unemployed	207	51.5
Retired/homemaker	49	12.2
Smoking status	-	-
Never	81	20.1
Former	169	42.0
Current	152	37.8
Time since curative colon cancer surgery	-	-
<6 months	98	24.4
6-12 months	183	45.5
>12 months	121	30.1
Presence of any chronic medical condition	-	-
Yes	106	26.4
No	296	73.6
History of any other major surgery	-	-
Yes	144	35.8
No	258	64.2
Family history of cancer	-	-
Yes	159	39.6
No	243	60.4

Table 2 indicates that both the Kolmogorov-Smirnov and Shapiro-Wilk tests suggest that the QoL variables are generally not normally distributed (p < 0.001). The distributions were positively skewed, with skewness values ranging from 2.620 (functional well-being) to 6.033 (additional concerns). Despite these violations, it was assumed that parametric tests could be used because of the large sample size (N = 402).

**Table 2:** Tests of normality for quality-of-life variables (N = 402) p < .001 indicates significant deviation from normal distribution among variables; N = 402. Although the Shapiro-Wilk and Kolmogorov-Smirnov tests indicated deviations from normality for all variables (all p <0.001), parametric tests were employed given the large sample size (N = 402) and the robustness of these tests with large samples. Skewness values ranged from 2.620 (functional well-being) to 6.033 (additional concerns), indicating positively skewed distributions

Variables	Kolmogorov-Smirnov			Shapiro-Wilk		
	Statistic	df	p	Statistic	df	p
Physical well-being	.227	402	<0.001	.701	402	<0.001
Social well-being	.285	402	<0.001	.493	402	<0.001
Emotional well-being	.399	402	<0.001	.565	402	<0.001
Functional well-being	.258	402	<0.001	.750	402	<0.001
Additional concerns	.260	402	<0.001	.558	402	<0.001

Table 3 shows that all QoL dimensions had a positive relationship. There were significant positive relationships between physical well-being and social well-being (r = 0.284, p < 0.01), emotional well-being (r = 0.110, p < 0.05), functional well-being (r = 0.373, p < 0.01), and additional concerns (r = 0.145, p < 0.01). On the same note, social well-being had significant relationships with emotional well-being (r = 0.307, p < 0.01), functional well-being (r = 0.394, p < 0.01), and additional concerns (r = 0.173, p < 0.01). Functional well-being (r = 0.443, p < 0.01) and additional concerns (r = 0.103, p < 0.05) correlated with emotional well-being. There was also a significant correlation between functional well-being and additional concerns (r = 0.318, p < 0.01). On balance, these findings indicate that a positive change in one domain of QoL is associated with positive changes in the other domains, in a consistent positive manner.

**Table 3:** Pearson correlations among quality-of-life dimensions (N = 402) N = 402; * = p < 0.05, ** = p < 0.01 were considered statistically significant Values represent Pearson correlation coefficients (r) between continuous variables

Variables	r	p
Physical well-being ↔ social well-being	.284**	< .001
Physical well-being ↔ emotional well-being	.110*	.027
Physical well-being ↔ functional well-being	.373**	< .001
Physical well-being ↔ additional concerns	.145**	.004
Social well-being ↔ emotional well-being	.307**	< .001
Social well-being ↔ functional well-being	.394**	< .001
Social well-being ↔ additional concerns	.173**	.001
Emotional well-being ↔ functional well-being	.443**	< .001
Emotional well-being ↔ additional concerns	.103*	.039
Functional well-being ↔ additional concerns	.318**	< .001

Table 4 shows that there were no significant differences in QoL dimensions by smoking status (all p > 0.05). Physical, social, emotional, and functional well-being and additional concerns showed very slight differences among never, former, and current smokers, with very minor effects (η 2 between 0.000 and 0.006), indicating that smoking status did not affect QoL in this sample.

**Table 4:** One-way ANOVA: quality-of-life dimensions by smoking status (N = 402) ANOVA: analysis of variance; SD: standard deviation Values are presented as mean ± standard deviation; group sizes are shown as N (%); Reported statistics include F-values, p-values, and effect sizes (eta-squared); p < .05 was considered statistically significant; N = 402. No statistically significant differences were found

Variable	Never (N = 81; 20.1%)	Former (N = 169; 42.0%)	Current (N = 152; 37.8%)	F	p	η²
	M ± SD	M ± SD	M ± SD			
Physical well-being	14.20 ± 1.13	14.33 ± 1.24	14.41 ± 0.85	0.995	.371	.005
Social well-being	14.02 ± 0.99	14.15 ± 1.95	14.21 ± 1.81	0.301	.741	.002
Emotional well-being	11.98 ± 0.72	12.18 ± 1.40	12.09 ± 1.25	0.811	.445	.004
Functional well-being	14.28 ± 0.87	14.53 ± 1.46	14.48 ± 0.98	1.170	.311	.006
Additional concerns	17.91 ± 2.32	17.85 ± 1.72	17.82 ± 0.99	0.082	.922	.001

Table 5 indicates that the majority of QoL dimensions were not significantly different across age groups (p > 0.05), but functional well-being showed significant differences, F (3,398) = 4.272, p = 0.006, eta = 0.031. Functional well-being (M = 15.20) was higher in participants aged >60 years than in younger age groups, whereas physical, social, emotional well-being, and additional concerns had no significant age effect.

**Table 5:** One-way ANOVA: quality-of-life dimensions by age group (N = 402) ANOVA: analysis of variance; SD: standard deviation Reported statistics include F-values, p-values, and effect sizes (eta-squared); ** = p < .01 was considered statistically significant; N = 402. Functional well-being showed a statistically significant difference across age groups, F (3, 398) = 4.272, p = .006, η² = .031, with the >60 age group reporting higher functional well-being (M = 15.20) compared to the other groups

Variable	≤40 years (N = 71; 17.7%)	41-50 years (N = 229; 57.0%)	51-60 years (N = 72; 17.9%)	>60 years (N = 30; 7.5%)	F	p	η²
	M ± SD	M ± SD	M ± SD	M ± SD			
Physical well-being	14.48 ± 1.58	14.24 ± 0.98	14.42 ± 0.88	14.50 ± 0.82	1.421	.236	.011
Social well-being	14.10 ± 1.97	14.22 ± 1.95	14.03 ± 0.93	14.03 ± 0.72	0.299	.826	.002
Emotional well-being	12.07 ± 0.83	12.10 ± 1.35	12.10 ± 1.18	12.27 ± 1.26	0.195	.900	.001
Functional well-being	14.41 ± 1.02	14.39 ± 1.20	14.42 ± 0.88	15.20 ± 1.79	4.272**	.006	.031
Additional concerns	17.62 ± 1.15	17.90 ± 1.72	17.78 ± 0.83	18.23 ± 2.94	1.132	.336	.008

Table 6 indicates that physical well-being (β = 0.251, p < 0.001), emotional well-being (β = 0.335, p < 0.001), social well-being (β = 0.188, p < 0.001), additional concerns (β = 0.209, p < 0.001), and age (β = 0.096, p = 0.016) were significantly associated with functional well-being. The model explained 38.4% of the variance in functional well-being (adjusted R² = 0.384), indicating a moderate explanatory capacity.

**Table 6:** Multiple linear regression predicting functional well-being from psychological and demographic variables (N = 402) R = .627; R² = .393; adjusted R² = .384; F (5, 396) = 51.818. Statistical significance is indicated as follows: *p < .05, **p < .01

Predictor	B	SE	β	t	p	95% CI (LL–UL)
Constant	1.704	0.862	—	1.977	0.049	[0.010, 3.397]
Physical well-being	0.276**	0.045	0.251	6.111	<0.001	[0.187, 0.365]
Emotional well-being	0.324**	0.040	0.335	8.088	<0.001	[0.245, 0.403]
Social well-being	0.128**	0.030	0.188	4.343	<0.001	[0.070, 0.187]
Additional concerns	0.152**	0.029	0.209	5.197	<0.001	[0.094, 0.209]
Age	0.144*	0.060	0.096	2.421	0.016	[0.027, 0.261]

## Discussion

This research paper explored how functional status and patient-centred variables are associated with determinants of postoperative well-being in patients undergoing curative resection for colon cancer, particularly in QoL domains. These findings have shown the interdependence among physical, emotional, social, and functional well-being in this population, as well as the significance of functional capacity as a key determinant of postoperative recovery and functional outcomes.

The current study revealed that physical well-being had a moderate relationship with both functional and social well-being, and a less significant, but significant relationship with emotional well-being and other concerns. These results are consistent with other available evidence suggesting that improved physical health or physical activity is more closely related to daily functioning and social participation than to the emotional aspects of QoL [13]. Social well-being in the current study exhibited moderate relationships with both emotional and functional well-being. In contrast, emotional well-being had the strongest relationship with functional well-being, which is essential for everyday functioning. These results are in line with the latest evidence showing that social capital/emotional health are interconnected with functional health status and overall QoL [14]. In our analysis, other areas of concern were weakly to moderately related to all QoL areas, with the highest relationship to functional well-being, indicating that QoL is multidimensional. This aligns with previous literature, which identified correlated physical, social, psychological, and spiritual QOL domains [15]. While the regression model explained 38.4% of the variance in functional well-being, a substantial proportion of variability remains unaccounted for. This may be attributable to factors not included in the model, such as tumour-related characteristics (e.g., cancer stage, disease severity), treatment-related variables (e.g., type of surgery, adjuvant therapy), and broader patient-related factors (e.g., nutritional status, socioeconomic conditions, access to postoperative care). These unmeasured variables may play an important role in shaping postoperative functional outcomes and should be considered in future research.

This sample showed no significant difference in QoL scores across smoking status groups, which is contrary to the established negative effects of smoking on pulmonary functioning and recovery following surgery [16]. Several factors might explain this finding. To begin with, the large percentage of ex-smokers (42.0%) might have diluted any visible difference between groups, since the effect of smoking cessation is associated with an incomplete recovery of functional and respiratory status with time. Second, smoking status was self-reported, and this may result in the under-reporting of smoking status due to social desirability bias, especially in a clinical setting. Additionally, the cross-sectional design captures outcomes at a single time point and may not reflect the cumulative or long-term impact of smoking on postoperative QoL. Although some studies have reported no significant differences in overall QoL between smokers and nonsmokers, with variations limited to specific domains such as satisfaction with health [17], these findings remain context-dependent. Overall, these factors may have contributed to the lack of statistically significant differences observed in this study.

In our research, physical, social, and emotional well-being did not differ with age, but functional well-being was higher in older adults. This is consistent with SNAC-K study findings indicating that age-related functional ability is preserved, with increased psychological and social well-being [18]. Additional concern scores were also relatively stable across age groups, which is consistent with previous studies indicating that these aspects of QoL do not depend on age [19].

In general, functional well-being in the present research was affected by a variety of factors, including emotional, physical, social, and more general psychosocial factors, indicating its multidimensionality. This result is consistent with earlier studies showing that greater well-being, robust social support, and age-related psychosocial resources are correlated with improved functional outcomes [13-15,18].

Together, these results add to the growing body of literature indicating that patient-reported functional status and QoL scales are useful prognostic variables alongside conventional clinical and tumour-related variables. The systematic monitoring of functional and psychosocial well-being during postoperative care can identify patients at risk of poorer functional status and offer multidisciplinary interventions that address aspects of QoL.

Limitations

This research has several limitations that must be considered when interpreting the findings. To begin with, the cross-sectional design does not allow causal inferences regarding the relationships between functional status, comorbidities, and QoL outcomes. Longitudinal follow-up would be required to ascertain the effect of changes in functional well-being on long-term survival. Second, convenience sampling was applied, which could cause selection bias and restrict the extrapolation of the findings to all patients who have curative surgery on colon cancer, especially those who are treated in other healthcare facilities. Third, the postoperative well-being was measured indirectly (QoL and functional outcomes) rather than as objective survival measures (overall or disease-free survival). Moreover, self-reported questionnaires were used to collect data, which are susceptible to recall and social desirability bias. The regression model did not include tumour-specific variables (e.g., cancer stage, treatment modality, or adjuvant therapy), which may have introduced residual confounding and influenced the observed associations. Lastly, the use of English-language questionnaires, even with the help of bilingual interviewers, may have limited participation among individuals with lower literacy levels or who are rural or socioeconomically disadvantaged. Also, screening out individuals with communication problems could have contributed to the underrepresentation of these groups. This may introduce selection bias and limit the extrapolation of results, specifically to the social determinants of health in the general population.

Future directions

A longitudinal research design should be used in the future to assess the predictive value of functional status and QoL domains for long-term survival and recurrence in patients with colon cancer. Causation would be enhanced by incorporating objective clinical endpoints and patient-reported measures. The relationships between functional status, comorbidity burden, tumour characteristics, and treatment modalities should also be further studied to develop holistic risk prediction models. There is also a need to conduct interventional studies to determine the impact of targeted physical, psychological, and social interventions on functional well-being and postoperative outcomes. Further studies that incorporate diversity in populations and healthcare settings would maximise external validity and provide region-specific evidence to inform clinical practices in low- and middle-income countries.

## Conclusions

This study demonstrates that physical, emotional, and social factors are significantly associated with functional well-being among patients with colon cancer following curative resection. The findings highlight the multidimensional nature of postoperative well-being, with emotional and physical well-being emerging as key correlates of functional status. These results underscore the importance of integrating routine functional and QoL assessments into postoperative care and survivorship planning. A multidisciplinary, patient-centred approach that addresses both physical and psychosocial needs may help improve overall postoperative well-being in this population.
